# Incorporating Dis/ability Studies and Critical Race Theory to combat systematic exclusion of Black, Indigenous, and People of Color in clinical neuroscience

**DOI:** 10.3389/fnins.2022.988092

**Published:** 2022-09-08

**Authors:** Teresa Girolamo, Termara C. Parker, Inge-Marie Eigsti

**Affiliations:** ^1^Department of Psychological Sciences, University of Connecticut, Storrs, CT, United States; ^2^Interdepartmental Neuroscience Program, Yale School of Medicine, New Haven, CT, United States

**Keywords:** underrepresentation, clinical neuroscience, BIPOC, DisCrit Theory, advocacy

## Abstract

This article reviews some of the ideological forces contributing to the systematic exclusion of Black, Indigenous, and People of Color (BIPOC) in clinical neuroscience. Limitations of functional near-infrared spectroscopy (fNIRS) and other methods systematically exclude individuals with coarse or curly hair and darker skin. Despite these well-known limitations, clinical neuroscience manuscripts frequently fail to report participant race or ethnicity or reasons for excluding participants. Grounding the discussion in Dis/ability Studies and Critical Race Theory (DisCrit), we review factors that exacerbate exclusion and contribute to the multiple marginalization of BIPOC, including (a) general methodological issues, (b) perceptions about race and disability, and (c) underreporting of methods. We also present solutions. Just as scientific practices changed in response to the replication crisis, we advocate for greater attention to the crisis of underrepresentation in clinical neuroscience and provide strategies that serve to make the field more inclusive.

## Introduction

The systematic exclusion of Black, Indigenous, and People of Color (BIPOC) in clinical research is a longstanding problem, despite awareness (Durkin et al., 2015), empirical evidence (Henrich et al., 2010), and calls to action (Maye et al., 2021). Insufficient efforts to include BIPOC with disabilities (Annamma et al., 2013) and inconsistent reporting practices (Choy et al., 2021) reinforce the underrepresentation of already minoritized individuals – that is, they multiply marginalize BIPOC from clinical populations. This crisis is exacerbated by limitations in the technical and methodological features of neurotechnology (Parker and Ricard, 2022; Webb et al., 2022). These limitations also hinder reproducibility and generalizability (Open Science Collaboration, 2015), as well as the translation of scientific findings into clinical applications and interventions. The limited evidence base regarding BIPOC can only be addressed when neuroscience as a field, and individual scientists, make a concrete commitment to reversing exclusion and increasing diversity (Wilton et al., 2020). This manuscript reviews current limitations to methodology, recruitment, and reporting practices in clinical neuroscience and offers solutions.

Dis/ability Studies and Critical Race Theory (DisCrit) describes race and dis/ability as social constructs that primarily involve not the individual differences themselves, but rather, how *others* respond to those individual differences (Annamma et al., 2013, 2016). This theory centers external perceptions about race and disability as impacting the experiences of marginalized individuals (Annamma et al., 2018), with intersecting identities giving rise to multiple marginalization (Crenshaw, 1991). For example, Black children in the United States are under-identified as having speech/language impairments (Robinson and Norton, 2019); at the same time, Black children are also over-identified and misdiagnosed with conduct disorder rather than autism (Mandell et al., 2007). The perceptions of others (in this example, clinicians) about race reinforce perceptions about disability (and vice versa), leading to negative outcomes. DisCrit conceptualizes inequity at the intersection of race and dis/ability (Annamma et al., 2013), paralleling the intertwined fight for civil rights and dis/ability rights in the United States and reflecting everyday realities (Turnbull et al., 2006) (i.e., a Black autistic individual is not *only* Black or *only* autistic, but rather, navigates daily life as someone others perceive as Black and autistic).

### Methodological limitations of neurotechnology for Black, Indigenous, and People of Color from clinical populations

#### Functional magnetic resonance imaging and functional near-infrared spectroscopy

Functional neuroimaging tools have led to dramatic advances in the diagnosis and study of communication disorders (Butler et al., 2020). Functional magnetic resonance imaging (fMRI) provides millimeter-level anatomical information, and also permits the assessment of regions of activation associated with an online behavioral task. While this information is highly informative, fMRI requires participants to remain in a confined space with little to no head movement, potentially eliciting anxiety and discomfort. MRI also involves significant environmental noise, which can be difficult to tolerate (Crosson et al., 2010). Hence, individuals from clinical populations whose sensory needs, anxiety, or difficulty in comprehending the need to remain motionless, are less able to participate in MRI studies; this includes individuals with neurodevelopmental disorders and cognitive impairment. Consequently, fMRI studies are more likely to include individuals with age-appropriate neurocognitive skills, and fewer neurodevelopmental disorder traits (Cosgrove et al., 2022).

Functional near-infrared spectroscopy uses the absorption of near-infrared light to measure hemodynamic oxyhemoglobin and deoxyhemoglobin concentrations in the cortex as a proxy for direct neural responses, similar to fMRI’s BOLD signal (Jöbsis, 1977; Ferrari and Quaresima, 2012; Scholkmann et al., 2014). NIRS is more robust than MRI to head and body motion; it also permits data collection in an unrestricted environment, avoiding the need to remain motionless in a small scanner bore. Thus, fNIRS permits the assessment of neural responses in a broader range of individuals, such as those with speech/language impairments (Butler et al., 2020).

The efficacy of fNIRS (and the methodologically similar electroencephalography, EEG) varies by melanin and hair type (Yücel et al., 2021). NIRS and EEG require adequate contact with the scalp for good signal reception, and the MRI head coil does not fit individuals with large afro-textured hair, nor does it allow for data collection in individuals with hair extensions, as many use metal (Parker and Ricard, 2022; Webb et al., 2022). Thus, as currently deployed, these important neuroscience tools are less effective with coarse and/or curly hair and with darker skin. Given the multiple challenges of data collection, researchers may explicitly or implicitly exclude BIPOC by screening them out; even when BIPOC are included, their hemodynamic responses may be less usable or make BIPOC look less responsive to stimuli (Yücel et al., 2021; Webb et al., 2022). These methodological challenges lead to the systematic and disproportionate exclusion of BIPOC individuals from neuroimaging research.

#### Potential solution: Interdisciplinary approaches

Ignorance about systematic exclusion leads to an evidence base that is biased and unrepresentative. To counter marginalization of BIPOC from clinical populations (Annamma et al., 2018), we must transform both the scientific process and neuroimaging methods, prioritizing the collection of high-quality data from diverse participants. A The New York Times editorial suggested that effective strategies to address scientific, technological, ecological, political, and economic challenges, such as water use and conservation, require interdisciplinary thematically organized problem-focused programs including stakeholders (Taylor, 2009). We endorse this “all-in” approach with thematically organized approaches to dis/ability and race in neuroscience. For example, Parker and Ricard (2022) called for researchers, engineers, Black hairstylists and barbers, and research participants to co-develop accommodations for diverse hairstyles. Additional participants in the larger effort would include BIPOC community members (Lewis and Oyserman, 2016; Maye et al., 2021), policymakers and commercial organizations (National Institutes of Health, 2021), legal and educational theorists to generate models of underrepresentation (Powell, 2012; Annamma et al., 2013), and psychometricians to develop analytical approaches using intersectionality theory (Bauer et al., 2021).

At a broader level, funding agencies, as the National Institutes of Health (2021) has done, must promote interdisciplinary calls for proposals to develop, implement, and disseminate evidence-based practices to combat structural systemic racism. The effectiveness of diversity initiatives must also be benchmarked to funding outcomes (Wilton et al., 2020). In the United States, Black PIs – who are more likely than white PIs to propose doing BIPOC- and community-related research – are less likely than white PIs to receive major NIH (R01) grants (Ginther et al., 2016; Chen et al., 2022). Yet current interdisciplinary initiatives reflect the leadership of BIPOC in the quest to improve scientific innovation and discovery by making neuroscience inclusive; thus, mitigating inequity in grant funding is of paramount importance. For instance, Yücel and colleagues are investigating the effects of hair type and skin pigmentation on the signal quality of fNIRS via a partnership with industry, as well as autism and linguistic researchers (Facebook Research, 2021). Another team, led by Etienne et al. (2020), developed inclusive EEG electrodes for Black individuals and other persons with coarse and curly hair. These approaches are consistent with federal funding priorities of improving minority health and promoting collaborative science (National Institutes of Health, 2021).

### Collective response to race and disability

#### Perpetuating issues impeding inclusive research

Given these and other limitations, BIPOC from clinical populations may be less likely to participate in neuroscience studies. Sampling practices, communication, and teaching can create a feedback loop that normalizes and perpetuates the systematic exclusion of such individuals from science. Over time, scientific practices can reify biased assumptions about race, dis/ability, and who can be included in research. In turn, these assumptions shape the development of research questions and recruitment methods, and impact future science via the training of junior scientists. Following DisCrit (Annamma et al., 2013), this cycle contributes to bias in the evidence base and in who is served by research practices (Lewis and Oyserman, 2016).

##### Convenience sampling and attrition

**FIGURE 1:**
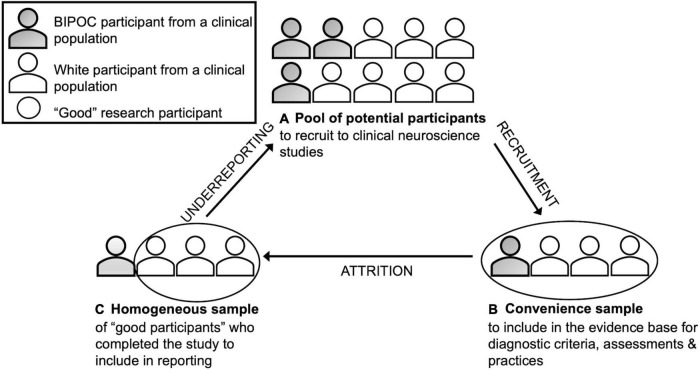
Application of Dis/ability Studies and Critical Race Theory (Annamma et al., 2018) to clinical neuroscience.

In clinical neuroscience, researchers recruit from a pool of participants who share a trait (e.g., autism plus language impairment); see Figure 1A. Researchers make assumptions about who is likely to contribute usable data and complete all study activities; such assumptions may exacerbate underrepresentation (Joseph and Dohan, 2009). Though they may aim for a sample that is representative of the population in terms of race, ethnicity, and other relevant variables (National Institutes of Health, 2017), time pressures on publications, grant applications, and career advancement, may lead to convenience sampling, which selects against BIPOC from clinical populations (Kasari et al., 2013; Durkin et al., 2015); see Figure 1B. As noted in “Functional magnetic resonance imaging and functional near-infrared spectroscopy”, assumptions about who is likely to generate usable data (e.g., white participants with age-appropriate cognitive abilities (Cosgrove et al., 2022); can further increase the underrepresentation of BIPOC from clinical populations.

Underrepresentation means there is limited information on how to engage BIPOC from clinical populations in research, which requires being sensitive to the intersection of race and disability (Maye et al., 2021). For instance, nearly all (94%) autism studies exclude individuals with intellectual disabilities, but studies typically do not report information on intelligence or limitations to generalizability (Russell et al., 2019). Considering that researchers are less likely to approach BIPOC than white individuals as potential participants (Wendler et al., 2005), it is likely that autistic BIPOC with intellectual disabilities are even more underrepresented. Furthermore, recruitment and research methods, such as a failure to make time to build rapport, can affect study completion. For example, an autistic BIPOC young adult may initially consent to participation, but grow uneasy in an unfamiliar setting (e.g., laboratory) with unfamiliar people who do not have ties to their community, and complete the study activities in a way that increases noise in their data. Thus, even when well-intentioned researchers recruit and run BIPOC, and when data are collected, the usable data may come primarily from a less diverse, mostly white, sample (Webb et al., 2022). Underreporting of research methods can mask relevant details about the initial pool of potential participants and those participants whose data is included in the final report, resulting in bias; see Figure 1C.

#### Potential solution: Participatory methods

Mitigating underrepresentation may require researchers to share power in the research process. In community-based participatory research (CBPR), researchers develop partnerships with community stakeholders to develop research questions, methods, and studies, that benefit all parties (Ellis et al., 2021). For research with BIPOC from clinical populations, such partnerships are practical and ethical. Community advisory boards guide and hold researchers accountable for the responsible conduct and dissemination of research (Ellis et al., 2021). Such partnerships align with self-advocacy movements, which advocate for research that reflects their priorities (Gowen et al., 2019). Community partners can advocate for particular outcomes, such as the translation of study findings into policy recommendations, directions for clinical practice, and development of supports. Ultimately, participatory research can help change the collective response of clinical neuroscience to race and disability.

A first step is to identify and remove barriers to participation. In addition to logistical factors (e.g., scheduling studies after work hours and on weekends), Black families and BIPOC overall report distrust of research (George et al., 2014; Shaia et al., 2020). Researchers should spend time building trust, either with community advisory boards or community organizations, on community terms (Ellis et al., 2021). In addition, we should consider how perceptions of disability and race (and the subsequent experiences of individuals) along with systematic exclusion from research as both participants and researchers can influence a participant’s comfort and subsequent performance (Shaia et al., 2020; Yücel et al., 2021). To mitigate that discomfort, researchers could plan a step by step preview of study activities with community partners prior to data collection to ensure activities are accessible to BIPOC from clinical populations.

### Underreporting of participant demographics

Underreporting of participant demographics, though common practice in neuroimaging (Choy et al., 2021; Goldfarb and Brown, 2022), contributes to bias. Our team is currently performing a systematic review of the reporting of sociodemographics in empirical, refereed fNIRS studies of speech and language impairments. These studies frequently fail to report race, ethnicity, and other demographics (e.g., socioeconomic status). Failure to report participant race and ethnicity constitutes colorblindness (Webb et al., 2022) and masks the true extent and nature of bias; the information necessary to understand variability is treated as irrelevant.

#### Potential solution: Reporting, interpretation, and use of research studies

To develop a more authentic evidence base, scientists should implement replicable reporting standards, which should have downstream effects on the interpretation and use of findings to develop studies and make decisions about the state of the science (Kane, 2012). Though responsible reporting cannot address the systemic exclusion of BIPOC from clinical populations from research, it can enhance reproducibility and transparency (Sabik et al., 2021). Per the American Psychological Association (2020) and the American Medical Association (Flanagin et al., 2021), race and ethnicity are social constructs, meaning that authors should report: (a) race and ethnicity together with other factors known to intersect with race and ethnicity; (b) the method by which race and ethnicity information was collected, and why (e.g., to respect funding agency requirements); (c) specific or self-reported labels versus broad categories for race and ethnicity (e.g., allowing people to self-report or select “Naxi” versus “Asian”); and (d) reasons for attrition, considering that some participants are more likely to be excluded than others. Best practices include reporting ethnicity, recognizing that the ethnicity of participants may differ from the ethnicity of researchers (Yücel et al., 2021).

In addition to race and ethnicity, reporting participant characteristics relevant to understanding the generalizability of the findings within that clinical population (e.g., social communication impairment, nonverbal intelligence) can increase our understanding of generalizability. For example, autistic BIPOC with co-occurring diagnoses are often excluded, such that our current understanding of autism is based primarily on white individuals without intellectual disability or language impairment (Durkin et al., 2015; Bottema-Beutel et al., 2021). Importantly, because there is no one-to-one correspondence of race and ethnicity with hair type or skin tone, collecting and reporting measures relevant to skin tone and hair type (e.g., level of skin pigmentation and hair density) may also informative (Facebook Research, 2021).

Researchers should be precise in their interpretation of research findings. Data from neuroscience experiments constitute just one piece of evidence; the scientific community should interpret and use that evidence in a fair and equitable manner, which may necessitate collecting further evidence to support the validity of study findings (Messick, 1989; Kane, 2012; Girolamo et al., 2022). In the case of BIPOC – and especially BIPOC from clinical populations – this entails the following steps: (a) critically asking what demographic and identity variables are necessary to understand representativeness; (b) asking whether participants in a study are representative of the population of interest; (c) deciding under what conditions study findings are or are not generalizable. Researchers should be equally precise in how they use study findings, whether from their or others’ work, to make decisions about the state of the evidence base. For instance, given that the quality of MRI signals is better in white participants with few neurodevelopmental disorder traits and age-appropriate intelligence (Cosgrove et al., 2022), the findings and methods of MRI studies may be less applicable to autistic BIPOC with intellectual disability.

## Discussion

The factors in underrepresentation of BIPOC from clinical populations in neuroscience are myriad, with DisCrit helping conceptualize such exclusion (Annamma et al., 2013). In addition to the solutions offered above, systems-level change is needed to make neuroscience more inclusive.

### Middle-out advocacy for systems change

As the leaders in research design, researchers inadvertently signal who is and is not welcome to participate (Lewis and Oyserman, 2016). As with fMRI (Cosgrove et al., 2022) and EEG (Choy et al., 2021), and fNIRS (Parker and Ricard, 2022), current neuroimaging practices insufficiently minimize racial, ethnic, and disability-relevant diversity, consistent with a model where individual differences are primarily a function of others’ reactions (Annamma et al., 2013). To mitigate exclusion, researchers must be proactive advocates for change. Funders of research, universities, and commercial organizations exert influence downward on researchers by deciding who and what to fund, publish, and promote (Janda and Parag, 2013). At the bottom of the research system are participants, who, unless they are part of a participatory partnership, only exert influence upward by electing to take part in research. Researchers are situated in the middle of this system. They mutually influence each another (e.g., when reviewing manuscripts and grants, thus shaping who and what is published or funded), but also exert upward influence on funders (e.g., when advocating for research or serving on a committee), and downward influence on participants and mentees (e.g., advising on research design, analysis, and reporting, and coaching students on best practices).

Within this structure, researchers are the only stakeholders who exert influence in three directions. Researchers are also the most knowledgeable about their studies and research practices. Thus, researchers are the best advocates for change in how research is conducted, evaluated, and funded. It is also critical to cite, center, and implement the suggestions of BIPOC researchers who bring light to these issues and generate solutions, such as community-based methods for autism research (Maye et al., 2021), develop inclusive fNIRS methods and tools (Etienne et al., 2020; Parker and Ricard, 2022; Webb et al., 2022), and present best reporting practices (Yücel et al., 2021). If clinical neuroscience researchers exert advocacy in these ways, there will be material changes in the valuation and funding of research, the scientific evidence base, and research culture.

## Conclusion

Overall, the self-perpetuating cycle of underrepresentation of BIPOC from clinical populations presents important challenges to the field of neuroscience. Using DisCrit as an explanatory pathway, this article discusses the factors exacerbating underrepresentation and outlines how researchers are uniquely positioned to effect change. It is our hope that researchers take up the call for advocacy and generate innovative solutions to make our field more authentically equitable and just.

## Data availability statement

The original contributions presented in the study are included in the article, further inquiries can be directed to the corresponding author/s.

## Author contributions

TG: conceptualization, methodology, formal analysis, investigation, writing – original draft, visualization, and supervision. TP: conceptualization, formal analysis, and writing – review and editing. I-ME: funding acquisition, project administration, and writing – review and editing. All authors contributed to the article and approved the submitted version.
